# An immunoinformatic strategy for developing peptide vaccines against autoimmune diseases by targeting cross-reactive bacterial antigens

**DOI:** 10.12669/pjms.42.3.13091

**Published:** 2026-03

**Authors:** Hamid Nawaz Tipu, Mustajab Alam

**Affiliations:** 1 Hamid Nawaz Tipu, FCPS, Department of Immunology, Armed Forces Institute of Pathology, Rawalpindi, Pakistan; 2 Mustajab Alam, FCPS, Department of Immunology, Armed Forces Institute of Pathology, Rawalpindi, Pakistan

**Keywords:** Autoimmunity, Bioinformatics, Human leukocyte antigen, Immunoinformatic pipeline, Peptide vaccine

## Abstract

**Objective:**

To identify potential immunogenic peptide vaccine candidates targeting bacterial proteins implicated in autoimmune disorders via molecular mimicry, employing a population-specific immunoinformatic approach.

**Methodology:**

This cross sectional in silico bioinformatics study was carried out in Department of Immunology, Armed Forces Institute of Pathology, Rawalpindi, in August 2025. Fourteen proteins from eleven bacteria were selected due to their reported association with autoimmune disorders. FASTA sequences were retrieved and 15mer peptides generated. Binding affinities to three commonest HLA II alleles in Pakistan were determined using NetMHCIIpan-4.0. Strong binders were screened with BLASTP to remove 100% homologous peptides to human proteins. Rest of the peptides were assessed for CD4 T lymphocytes immunogenicity using Immune Epitope Database combined method.

**Results:**

From 14 bacterial proteins of 11 bacteria, 240 initial strong binders to HLA DRB1*03:01, 13:01 and 14:04 were identified. Four were excluded due to complete homology to at least one of human proteins. Of the remaining 236, 38 peptides exhibited high CD4 immunogenicity score (median percentile rank < 20). These peptides originated from eight proteins of seven bacterial species, each associated with specific autoimmune disorders.

**Conclusion:**

The immunoinformatic approach successfully identified 38 peptides as potential candidates for synthetic peptide vaccines against bacterial proteins implicated in autoimmunity. It has advantage of being rapid, population specific and reduces cost. It should be kept in mind that all bioinformatic findings must be confirmed by in vitro functional assays and in vivo studies before clinical trials.

## INTRODUCTION

Autoimmune disorders, that are characterized by breakdown in self-tolerance, affect millions of people worldwide. The yearly increase in incidence and prevalence is estimated to be 19.1% and 12.5% respectively.^1^ The etiology of autoimmune disorders is multifactorial and it involves combination of genetics and environment. A leading factor in initiation of autoimmune cascade is molecular mimicry, whereby, our immune system confuses human proteins for bacterial proteins due to either structural homology or amino acid sequences similarity. This results in activation of T and B lymphocytes attacking human tissue instead of bacterial proteins. A wide range of bacteria and their structural proteins have been implicated in various autoimmune disorders. A common example is M protein of Streptococcus pyogenes cross reacting with cardiac myosin resulting in rheumatic fever and rheumatic heart disease.^2^

Conventional treatment strategies for autoimmune disorders include anti-inflammatory drugs, immunosuppressants and therapies targeting specific molecules.^3^ But each of these therapeutic modalities carries its demerits like opportunistic infections. Even latest treatments like combination antibodies, antigen specific immunotherapies, nanomaterials and mRNA vaccine techniques and hematopoietic stem cell transplantation (HSCT) have met limited success only. Limited therapeutic efficacy can be attributed to a host of factors like immune system heterogenity, safety concerns, toxicity related to treatment or trial design limitations.^4^ Until now, several trials have been carried out testing the efficacy of peptide vaccines against autoimmune disorders like multiple sclerosis, diabetes mellitus, rheumatoid arthritis and Guillain-Barré syndrome.^5^,^6^

Peptide vaccines serve a promising alternative to conventional therapy or vaccines because they utilize short synthetic peptide sequences tailored to specific needs like Human leukocyte antigen (HLA) binding, regulatory T lymphocytes stimulation and T cell receptor (TCR) or B cell receptor (BCR) binding.^7^ These vaccines act after the peptide antigen is internalized by dendritic cells where it is degraded into peptides and presented to TCR in complex with HLA, resulting in T cell stimulation.^8^ By enabling the design of precisely targeted immune interventions, these vaccines hold the potential to modulate immune responses without broadly suppressing immunity, thereby reducing treatment-associated risks. With the advent of bioinformatics, computational tools have significantly streamlined the process of peptide identification for vaccine design.^9^,^10^

In this article, using an immunoinformatic pipeline, that leverages bacterial proteome screening, HLA binding affinity determination and epitope prediction algorithms, we aim to identify the tolerogenic peptide vaccine candidates that can interrupt the molecular mimicry. This approach in future can aid in designing synthetic peptide vaccines for wet lab testing and clinical trials. However, it must be emphasized that ultimately such predictive *in silico* work needs to be confirmed through *in vivo* experiments. Experimental validation of such approaches can revolutionize how autoimmune disorders are prevented and treated, shifting from symptom suppression to immune tolerance restoration.

## METHODOLOGY

This cross-sectional in silico bioinformatics study was carried out in Department of Immunology, Armed Forces Institute of Pathology, Rawalpindi, Pakistan, in August 2025. The sample size included 240 15mer peptides from 14 proteins of 11 bacteria which have been commonly implicated in autoimmune disorders (see Table-I).^11^ The sampling technique was non-probability convenience sampling and being an in silico bioinformatic study encompassing peptide sequences from proteomic databases, sample size calculation was not applicable.

**Table-I:** Number of 15mer strong binders against HLA alleles DRB1*03:01, DRB1*13:01, DRB1*14:04.

S. No.	Bacteria	Protein	Accession No	Number of strong binders (n=240)	Proportion of strong binders (%)
1	*Streptococcus pyogenes*	M protein	>AAA26918.1	22	9.17
2	*Campylobacter jejuni*	LOS (Lipo-oligosaccharide)	>WP_438411866.1	20	8.33
3	*Klebsiella pneumoniae*	Pullulanase (PulA)	>STR29919.1	39	16.25
4	*Mycobacterium tuberculosis*	Heat Shock Protein 65 (HSP65)	>WOE54985.1	1	0.42
5	*Escherichia coli*	DNA gyrase	>WCP16997.1	13	15.83
		GroEL	>UXN76221.1	25	
6	*Borrelia burgdorferi*	OspA (Outer surface protein A)	>CAA32579.1	14	5.83
7	*Helicobacter pylori*	CagA,	>ACY01385.1	1	10.83
		Urease B subunit	>ABG73414.1	25	
8	*Chlamydia trachomatis*	HSP60	>AAC68350.1	18	7.5
9	*Yersinia enterocolitica*	Outer membrane proteins (YopH)	>AAN37546.1	10	4.17
10	*Salmonella typhimurium*	OmpC	>AIH09857.1	10	11.25
		SipC	>AAA75170.1	17	
11	*Shigella flexneri*	Invasion plasmid antigen B (IpaB)	>SVH88885.1	25	10.42

### Inclusion & Exclusion Criteria:

The FASTA sequence of 14 bacterial proteins was obtained from public database National Library of Medicine.^12^ Inclusion criteria were only those peptides that showed strong binding affinity with common HLA alleles in Pakistani population were included. The common HLA alleles were determined from our previously published data^13^ and binding affinity of 15mer peptides derived from FASTA sequence of proteins was determined using NetMHCIIpan-4.0 server employing artificial neural networks.^14^ Table-I shows number of 15mer strong binders against their proteins and bacterial names. Exclusion criteria included weak or non-binder 15mer peptides.

These strong binder 15mer peptides were progressed into further proteomic analysis using National Center for Biotechnology Information (NCBI) Basic Local Alignment Search Tool for Proteins (BLASTP).^15^ The purpose was to identify any 15mer peptide that displayed 100% homology to any of human protein from *Homo sapiens* (taxid:9606) protein database containing 408172754 sequences and remove it from downstream analysis. The remaining sequences were tested for CD4 cell immunogenicity with combined (seven allele and immunogenicity) method using Immune epitope database T cell prediction tools.^16^ Median percentile rank below 20 was used as cutoff for immunogenicity prediction.

### Statistical analysis:

Frequencies and percentages of strong binders were calculated using Microsoft Excel 2021. A one-way ANOVA was performed to compare the mean number of strong binder peptides across the bacterial species included in the study.

### Ethical approval:

Ethical approval was obtained from the Ethics Review Committee of the Armed Forces Institute of Pathology, Rawalpindi, Pakistan (Certificate No: IMM-1/READ/25/3929; dated June 20, 2025).

## RESULTS

A total of 14 proteins from 11 bacteria were included in study. After initial HLA binding affinities determination in NetMHCpanII-4.0, 240 15mer peptides were found to be strong binders to HLA alleles DRB1*03:01, 13:01 and 14:04. The number of strong binders against respective bacteria and protein is shown in Table-I. The one-way ANOVA analysis revealed no statistically significant difference in binder counts among species (F = 0.75, p = 0.682), indicating that the variation observed in peptide numbers is unlikely to be species-dependent. HLA alleles were selected based on top three commonest alleles in Pakistani population as determined by us previously, employing next generation sequencing.

**Fig.1:**
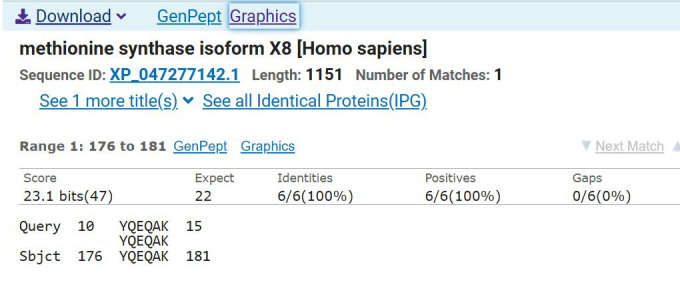
NCBI BLASTP showing query 10 peptide matching 100% identities with human methionine synthase.

**Fig.2:**
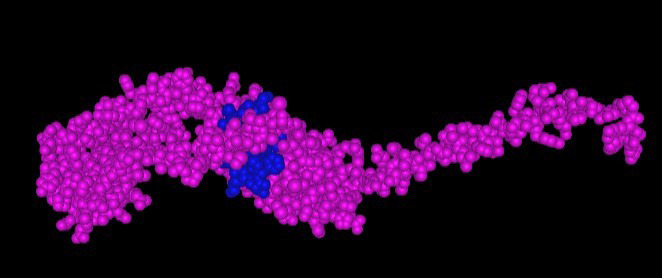
Sphere diagram of HLA DRB1*03:01(pink) in complex with peptide GRNVVLDKSFGAPTI (blue), docked in CABS-Dock and generated in icn3D.

After NCBI BLASTP analysis for removing peptides 100% homologous to human proteins, four peptides were removed. Peptides 1, 8, 9, and 10 (among 240) displayed 100% similarity to at least one of reference sequences and were removed from analysis. Fig.1 shows 100% matching of peptide sequence 10 with human protein methionine synthase at all 6/6 identities. This sequence was removed at this stage to avoid cross reactivity and subsequent possible autoimmunity. All these four peptides belonged to M protein from *Streptococcus pyogenes*. Remaining 236 peptides were tested for CD4 T cell immunogenicity prediction. Only 38 were found to be good immunogenic peptides having median percentile rank under 20. Table- II shows the good CD4-immunogenic peptides with their rank, protein, and bacterial origin. These 38 peptides are good candidates for synthetic peptide vaccines against respective bacteria. Fig.2 shows sphere diagram of HLA DRB1*03:01 in complex with a 15mer peptide. The docking of HLA DRB1*03:01 with peptide has been done in CABS-Dock server with flexible docking protocol and image has been generated in icn3D.

**Table-II:** 15mer SB peptides that are strongly immunogenic with their rank, protein and bacterial names.

S No	Bacteria	Protein name	Peptide	Median Percentile Rank
1	Escherichia coli	GroEL	GRNVVLDKSFGAPTI	9
			RNVVLDKSFGAPTIT	9
			KGRNVVLDKSFGAPT	9.4
			PKGRNVVLDKSFGAP	11
			PFILLADKKISNIRE	13
			SPFILLADKKISNIR	13
			VALIRVASKLADLRG	7.7
			GVALIRVASKLADLR	7.7
			GGVALIRVASKLADL	7.7
			ALIRVASKLADLRGQ	14
2	Chlamydia trachomatis	HSP60	PKIFVTDQKIHCLFP	17
			APKIFVTDQKIHCLF	17
			LVKGIQTQKGYRVPS	15
			VKGIQTQKGYRVPSF	14
3	Salmonella Typhimurium	OmpC	YTGGLKYDANNIYLA	17
			VYTGGLKYDANNIYL	17
			TGGLKYDANNIYLAA	16
4	Salmonella Typhimurium	SipC	RRKILMMRRLNLMPE	9.4
			SLNIYILSKRLESVE	15
			MRRKILMMRRLNLMP	8.7
			WYCRGVRAVRRYSGN	17
			PMRRKILMMRRLNLM	9
5	Klebsiella pneumoniae	pullulanase	GCINVIVRDGTNKLI	11
			CINVIVRDGTNKLID	15
			NVIVRDGTNKLIDSD	17
			INVIVRDGTNKLIDS	15
			QVELVIYSADKKVIA	6.7
			VELVIYSADKKVIAS	7.5
			ELVIYSADKKVIASH	11
			LVIYSADKKVIASHP	12
6	Helicobacter pylori	Urease B	ITNALIVDYTGIYKA	19
			DTHIHFISPQQIPTA	18
7	Campylobacter jejuni	Class E lipooligosaccharide biosynthesis protein	HSLKFFKKIRKHSMI	9.8
			IHSLKFFKKIRKHSM	11
			NGFILSNKNNEIMKI	17
			VNGFILSNKNNEIMK	19
8	Shigella flexneri	IpaB	EQALNPIMKAVIEPL	17
			QALNPIMKAVIEPLI	18

## DISCUSSION

In this study we used immunoinformatic analysis to identify potential peptide vaccine candidates from bacterial proteins implicated in autoimmune disorders via molecular mimicry. Our working pipeline included protein identification followed by amino acid sequence extraction, HLA II binding affinity determination with common local alleles, BLASTP homology screening and at the end, CD4 T cell immunogenicity prediction. We shortlisted 38 15mer peptides from 14 bacterial proteins as synthetic peptide vaccine candidates because these are strong HLA II binders, lack 100% homology to human proteins and are fairly immunogenic. This stepwise in silico approach not only accelerates vaccine candidate discovery but also reduces the costs, biosafety concerns, and ethical issues associated with early-stage wet-lab experimentation.^17^

Our results indicate that computational tools can significantly shorten peptide vaccine design whereby conventional vaccine approaches are hampered either by safety concerns or time constraints. Identification of 38 peptides from eight proteins of seven bacteria highlight diverse nature of this approach as vaccine candidates against a wide variety of autoimmune disorders, as shown in Table-II, have been identified.^18^,^19^ The population specific tailoring for only locally prevalent HLA alleles further adds specificity to peptides and can substantially influence vaccine efficacy. Population specific HLA databases can be used in other countries to identify strong binders to locally prevalent HLA alleles. Or binders to a large dataset of HLA alleles can be used to target a broader specificity vaccine though it may extensively burden the analysis servers. Previously we have used similar approach to identify peptide vaccine candidates against aspergillus, hepatitis C^9^ and CCHF^10^ proteins.^20^

Recently similar approaches have been used to design peptide vaccines against coronavirus during COVID epidemic.^21^-^23^ Wang B et al. have suggested peptide vaccines against cytokines like TNF alpha and interferon alpha to treat autoimmune disorders like systemic lupus erythematosus (SLE) or rheumatoid arthritis (RA).^24^ Weathington et al back in 2003 proposed slightly different approach to treat autoimmune disorders by targeting idiotypic antibodies or T cell receptors with complementary peptide vaccines.^25^

Another aspect of our study is targeting short synthetic peptides not exceeding 15 amino acids because these have fewer side effects than whole protein like in live attenuated vaccines. Shorter peptides also allow more controlled manufacturing processes, reduced risk of cross-reactivity, and better scalability for mass production. Moreover, their modular nature means they can be rapidly re-engineered to address newly emerging autoimmune triggers or adapted for combination therapy with other immune modulating agents. Our study provides a reproducible, population specific immunoinformatics approach for identifying tolerogenic peptide vaccine candidates against molecular mimicry driven autoimmunity offering a pathway towards safer and targeted immune modulation. Prioritizing precision over broad immunosuppression carries the potential to reduce treatment related morbidity, improve long term disease control and enhance life quality for autoimmune patients worldwide.

### Limitations:

In silico computational tools are not without some inherent limitations. Although these are quite sophisticated employing iterative algorithms but these cannot capture in vivo antigen processing and presentation in local cytokine milieu. Factors such as peptide stability in physiological conditions, post-translational modifications, and interaction with regulatory immune cells remain unpredictable in purely computational frameworks. Hence, in vitro assays using human cells and animal models are essential to validate the immunogenicity potential of synthetic peptides. Ultimately, a multi-stage validation process starting from in silico prediction, moving to in vitro functional assays, and culminating in controlled in vivo studies offers the most reliable path toward translating computational predictions into clinically viable peptide vaccines.

## CONCLUSION

The immunoinformatic pipeline provides a fast and population specific method to prioritize peptides against bacterial proteins implicated in molecular mimicry driven autoimmune disorders. The 38 peptides we identified represent good entrant for experimental validation and development of synthetic peptide vaccines. Future work should integrate structural modeling, in vitro functional assay and preclinical trials to confirm safety, immunogenicity and therapeutic potential before clinical use.

### Authors’ Contribution:

**HNT:** Conceived, designed, did data collection, manuscript writing, statistical analysis & editing of manuscript, is responsible for integrity of research.

**MA:** Did review and final approval of manuscript.
